# Supplementary material for: Effects of Resveratrol on Mouse B16 Melanoma Cell Proliferation through the SHCBP1-ERK1/2 Signaling Pathway
Source: Molecules. 2023 Nov 15;28(22):7614. doi: 10.3390/molecules28227614 (PMC10674768; doi:10.3390/molecules28227614)
Supplement: Supplementary file 1 [file molecules-28-07614-s001.zip › molecules-2646879-supplementary.pdf]

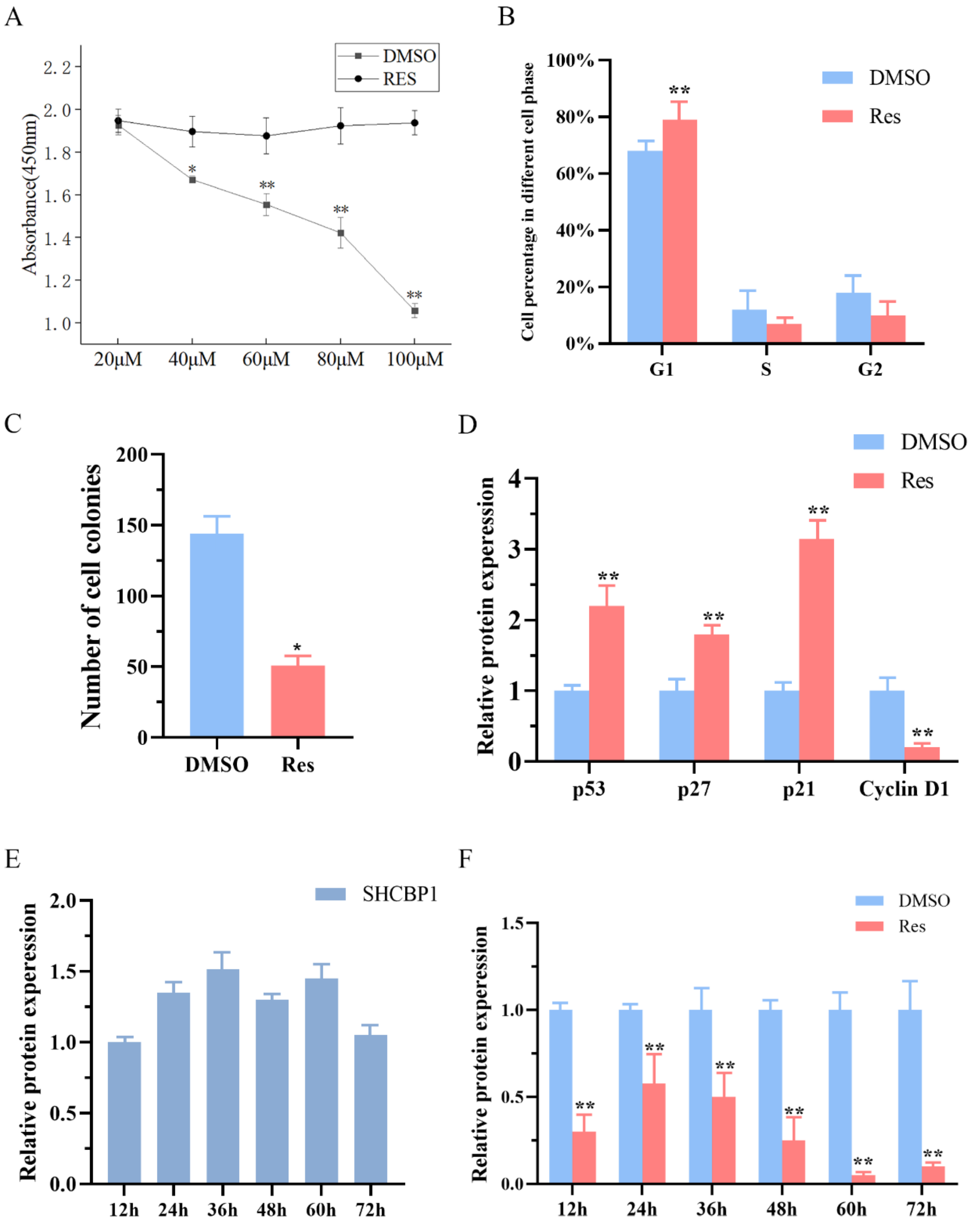

Figure S1. (A) Screening the concentration of resveratrol to inhibit B16 cells, *$p< 0.05$; **$p< 0.01$.(B) The number of G1, S and G2 cells in B16 cells was detected by flow cytometry, and DMSO was added as the control group. (C) Crystal violet fuel would be used to stain the subclonal cells formed after Res treatment for quantitative statistics of cell cloning, *$p< 0.05$;**$p< 0.01$.(D) The expression of p21, p27, p53 and Cycling D1 proteins in B16 cells was detected by western blotting for 48h after Res addition, *$p< 0.05$;**$p< 0.01$.(E,F) Protein was collected every 12h to detect the expression of SHCBP1 in B16 cells, *$p< 0.05$;**$p< 0.01$.

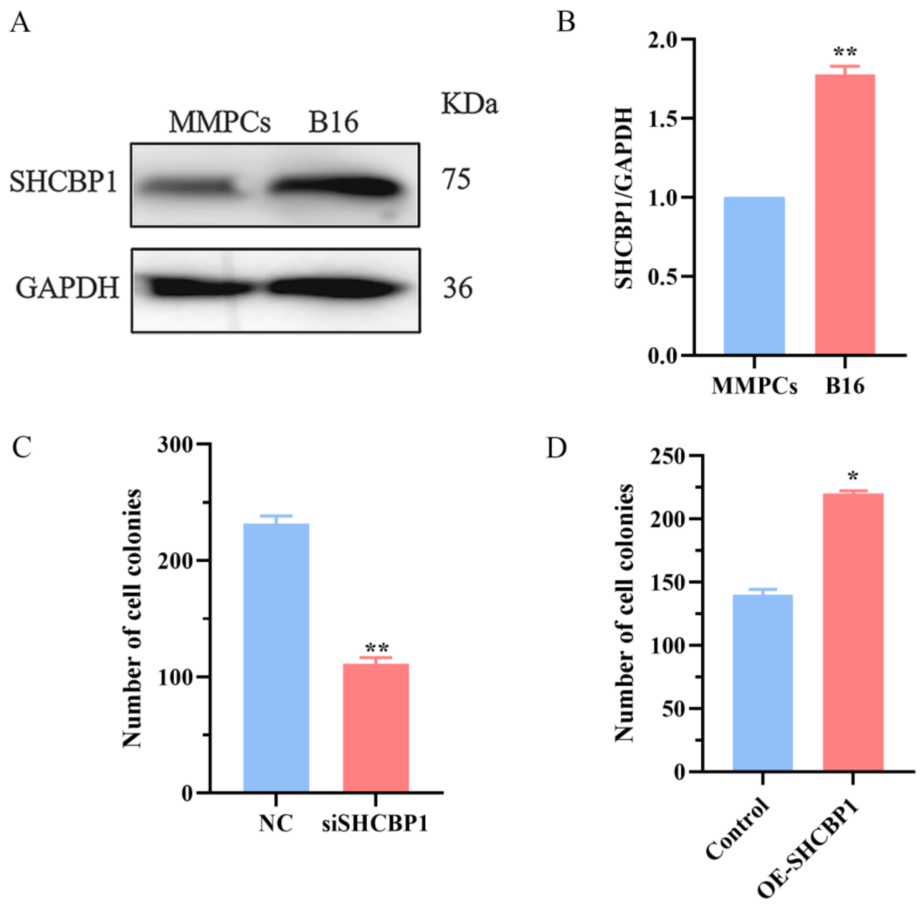

Figure S2. (A,B) Expression of SHCBP1 protein in MMPCs and B16 cells was detected. MMPCs: Mouse melanin precursor cells. *$p<0.05$;**$p<0.01$,n=3.(C)The clonal formation ability and quantification results of B16 cells were measured after continuous culture for 13 days. *$p<0.05$;**$p<0.01$.(D)The clonal formation ability and quantification results of B16 cells were measured after continuous culture for 7 days, *$p<0.05$;**$p<0.01$.
